# High-sensitive cardiac troponins and CK-MB concentrations in patients undergoing cardiac surgery

**DOI:** 10.1186/cc14238

**Published:** 2015-03-16

**Authors:** N De Mey, I Brandt, C Van Mieghem, K De Decker, G Cammu, L Foubert

**Affiliations:** 1 OLV AALST, Aalst, Belgium

## Introduction

Hs-cTn is the new standard cardiac biomarker for the diagnosis of myocardial necrosis. We conducted a prospective study to compare the course and values of the Hs-cTn and CK-MB after CABG and OPCAB. We also evaluated the relationship between values >10 × 99th percentile URL of CK-MB and Hs-cTn as a possible marker for perioperative myocardial infarction.

## Methods

All adult patients undergoing cardiac surgery between February and August 2014 were included. Exclusion criteria were urgent surgery, GFR <60 ml/minute/1.73 m^2^, CK-MB >4 μg/l and/or Hs-cTn >14 ng/l at baseline (BL). Hs-cTn and CK-MB were measured before induction (BL), upon arrival in the ICU and at fixed times after arrival. Patients with perioperative AMI as defined by the third universal definition of AMI were excluded for *post hoc *analysis [1].

## Results

**Figure 1:**
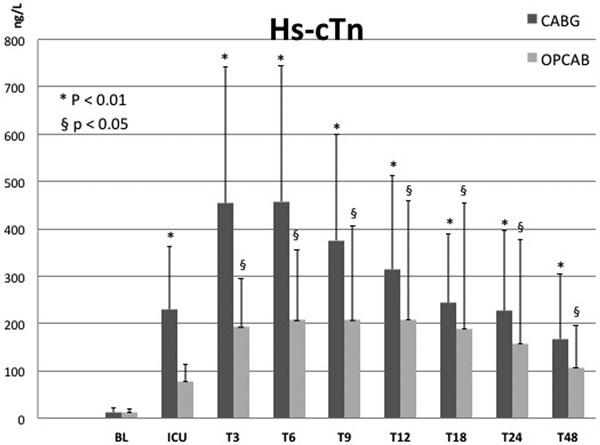


Of the 93 patients admissible for inclusion, 40 in the CABG and 14 in the OPCAB group met all inclusion criteria in this preliminary dataset. CK-MB values are higher from ICU arrival up to T24 versus baseline in both CABG and OPCAB (*P *< 0.0001) with a peak at T3. For Hs-cTn, ICU up to T48 values are higher (*P *< 0.01) in CABG with a peak at T6, and from T3 to T48 in OPCAB (*P *< 0.05) versus baseline (Figure 1). In CABG patients CK-MB levels are higher versus OPCAB from ICU up to T12 (*P *< 0.03), and from ICU to T48 for Hs-cTn levels (*P *< 0.02). In 39 CABG patients (97.5%) and 10 OPCAB patients (71.4%) all individual Hs-cTn values are above 140 ng/l (= 10 × 99th percentile of URL).

## Conclusion

Both CK-MB and Hs-cTn levels increase significantly after cardiac surgery. Postoperative Hs-cTn levels exceed the 10 × 99th percentile of URL in nearly all CABG patients. Our data show an important discrepancy between the 10 × 99th percentile for both biomarkers, and suggest that a different definition for postoperative AMI may be needed when Hs-cTn is used.
